# The Association of Waist Circumference with the Prevalence and Survival of Digestive Tract Cancer in US Adults: A Population Study Based on Machine Learning Methods

**DOI:** 10.1155/2022/2492488

**Published:** 2022-10-06

**Authors:** Xingyu Jiang, Qi Liang, Huanhuan Xu, Shouyong Gu, Lingxiang Liu

**Affiliations:** ^1^Department of Oncology, The First Affiliated Hospital of Nanjing Medical University, 300 Guangzhou Road, Nanjing 210029, China; ^2^Geriatric Institute, Jiangsu Province Geriatric Hospital, Nanjing Medical University Affiliated Geriatric Hospital, Nanjing 210029, China

## Abstract

**Aims:**

This paper aims to investigate the relationship of waist circumference (WC) with digestive tract cancer morbidity and mortality.

**Methods:**

Based on the data from a nationally representative US population survey, we summarized the prevalence of digestive tract cancer and all-cause mortality of cancer patients across WC quartiles. Adjusted logistic regression and restricted spline curve were used to analyze WC and the prevalence of digestive tract cancer. Moreover, Cox regression and the Kaplan-Meier curve were applied to investigate the association of WC with all-cause mortality. We also attempted to make a model to predict cancer happening.

**Results:**

This paper included a total of 34,041 participants, with digestive tract cancer observed in 265 (0.7%) individuals. WC was positively associated with digestive tract cancer morbidity after full adjustment of covariates (OR: 1.72 and 95% CI: 1.41-2.10). Also, individuals in the highest WC group had a higher risk of digestive tract cancer (Q4, OR: 2.71 and 95% CI: 1.48-5.00). Moreover, no significant association was observed in upper digestive cancer, and WC was associated with a longer survival time once diagnosed (hazard ratio (HR): 0.50 and 95% CI: 0.28-0.92). Finally, the model we made proved to be effective.

**Conclusion:**

High WC is a risk factor for digestive tract cancer with or without adjusting for body mass index, especially those located in the lower digestive tract. However, once digestive tract cancer has been diagnosed, patients with higher WC showed better survival outcomes. Moreover, machine learning methods can be used to predict digestive tract cancer risk in the future.

## 1. Introduction

Over the past decade, obesity has become a growing health threat worldwide, which is estimated to contribute to about 11.9% of cancer in males and 13.1% in females [1, 2]. Accumulating research had certificated that obese individuals were at an elevated of multiple digestive system cancer, including esophagus, gastric, colon, and rectal cancer [3–7].

Body mass index (BMI) is currently the foremost anthropometric index to evaluate the fat distribution of the body or the related health risk in most studies [8, 9]. However, obesity is a heterogeneous metabolic condition: Abdominal fat accumulation results in a more adverse obesity phenotype associated with worse metabolic profiles than subcutaneous accumulation. BMI alone is unable to capture the distribution of body fat or distinguish between adipose and muscle tissue [10]. A mildly elevated BMI (25-30 kg/m^2^) was reported to improve the survival of certain digestive tract cancer [11, 12]. Therefore, BMI is insufficient to fully understand obesity-related digestive tract cancer risk.

Among the several body measures, WC strongly correlates with abdominal fat distribution [13], and self-measurement of WC can be obtained easily. A recent consensus statement emphasized the importance of WC in clinical practice, given the advantages in stratifying obesity-related health risks than BMI [10, 14]. There used to be several studies revealing that WC had an adverse implication on all-cause death and cancer-related mortality [15–18]. Interestingly, after adjusting WC, BMI seemed to be a protective or neutral factor [10]. Instead, the clinical significance of WC can be fully demonstrated only after adjusting BMI [10, 19]. However, most evidence was from cardiovascular or metabolic diseases, and few studies investigated the association between WC and cancer prevalence with BMI adjusted for [20–24]. It remains unclear whether high WC could elevate the risk of digestive tract cancer.

Therefore, this article aims to analyze the relationship of WC with the prevalence and prognosis of digestive tract cancer.

## 2. Materials and Methods

### 2.1. Data Source

National Health and Nutrition Examination Survey (NHANES) is a publicly available database recording fit and nutrition conditions of adults and teenagers in the US (https://www.cdc.gov/nchs/nhanes/irba98.htm). The continuous NHANES data were collected in a 2-year cycle via face-to-face conversation and physical or laboratory examination. Our research used the 7 continuous NHANES data cycles, including (2001-2002, 2003-2004, 2005-2006, 2007-2008, 2009-2010, 2011-2012, and 2013-2014). Demographic characteristics, dietary, lifestyle factors, education levels, and medical conditions were collected via the conversation. Physical examinations, including weight, height, and WC, were measured in the mobile examination centers [25]. Moreover, the National Death Index (NDI) was used to investigate the association of all-cause mortality with digestive tract cancer patients. This database collects information about death or censoring by a medical examination (December 31, 2015).

All individuals aged ≥18 years with matched survival data were included, while those who were (1) without weight, height, or WC information (*n* = 2326) and (2) pregnant (*n* = 1081) were excluded from further analysis. NHANES followed the Health and Human Services policy and was approved by the National Center for Health Statistics. Sample participants were fully informed of the process and consented to participate in this survey.

### 2.2. Measurement of BMI and WC

BMI is calculated as the following equation: *BMI* = *weight* (*kilograms*)/*height* (*meters* *squared*). Next, BMI was divided into groups following the categories of World Health Organization (WHO): <18.5 kg/m^2^ is underweight, 18.5-24.9 kg/m^2^ is normal weight, overweight is defined as 25.0-29.9 kg/m^2^, class I obesity means BMI between 30.0 and 34.9 kg/m^2^, class II obesity means 35.0 and 39.9 kg/m^2^, and class III obesity means ≥40 kg/m^2^. Waist circumference was measured at the end of normal expiration in a standing position. It was measured above the uppermost lateral border of the right ilium, to the nearest 0.1 cm with the tape snug but not compressing the skin. The classification standard of WHO is between 94 and 101.9 cm for men and 80.0 and 87.9 cm for women, based on which abdominal obesity was defined as a WC greater than 102 in men and 88 in women [10]. More details are recorded in the Anthropometry Procedures Manual of NHANES (https://www.cdc.gov/nchs/data/nhanes/nhanes_07_08/manual_an.pdf).

### 2.3. Definition of Digestive Tract Cancer

In the face-to-face conversation, participators were asked the following two questions: (1) “whether they were ever told that they had cancer or malignancy” and (2) “which kind of cancer or malignancy they suffered by the health professionals.” In this paper, individuals with malignant tumors in the esophagus (*n* = 17, 0.04%), stomach (*n* = 26, 0.07%), colon (*n* = 217, 0.64%), and rectum (*n* = 12, 0.04%) were defined as patients with digestive tract cancer. Among them, small bowel cancer was not present in the dataset due to its low prevalence. Moreover, we stratified the digestive tract cancer into the upper and lower. Esophagus cancer and stomach cancer belong to the upper digestive tract cancer, and the rest belong to the other. These definitions were consistent with several epidemiological original types of research and one meta-analysis [26–28].

### 2.4. Covariates

Covariates were included by taking reference to the previous studies: age, gender (male or female), race (non- Hispanic Whites, non-Hispanic Blacks, Mexican Americans, other Hispanic, and other races), economic status (described as poverty-income ratio), and different education levels (not attended high school, high school, college, or above) [29, 30]. Diabetes history (diabetes, borderline diabetes, or nondiabetic) and whether they were smokers or alcoholics were collected using a health questionnaire during the conversation. Participants who smoked 100 cigarettes or above in their lifetime were recorded as smokers; participants who consumed 12 drinks or above per year were recorded as alcoholics [31]. Hemoglobin A1c (HbA1c) (mmol/L) and fasting plasma glucose (FPG) (mmol/L) were obtained in the laboratory, whereas the estimated glomerular filtration rate (eGFR) was calculated according to chronic kidney disease-epidemiology collaboration [32].

### 2.5. Statistical Analysis

First, we preprocessed the dataset via multiple imputations to fill missing values (except the outcome variable) to maximize statistical power and minimize deviation [33, 34]. We used the Kolmogorov-Smirnov test to test whether the continuous data is the normal distribution or skewed distribution. Normally distributed variables were presented as *mean* ± *standard* *deviation*, whereas skewed distributed variables were presented as median with Q1-Q3. Categorical variables were presented as percentages. Characteristics between those with or without digestive tract cancer risk were compared by one-way ANOVA analysis (normally distributed variables), Kruskal-Wallis test (nonnormal distributed variables), and chi-square test (categorical variables) as appropriate. Most cancer patients are elderly, so we separated the age as <80 and ≥80 to make the underlying association clearer. We used the adjusted logistic regression models to analyze the association between WC and digestive tract cancer risk, and the results were shown as OR with a 95% CI. In the minimally adjusted model, only old age, gender, smoking, drinking, and HbA1c were in adjustment. The BMI was additionally adjusted for the fully adjusted model. WC was also analyzed as four categories grouped by interquartile range in the regression model, setting the lowest group (55.5 to 86.8 cm) as the reference. In contrast, we performed a similar logistic regression analysis on BMI and the prevalence of digestive tract cancer. Moreover, a restricted cubic spline with 5 knots (5%, 25%, 50%, 72.5%, and 95%) was used to illustrate the relationship between WC and digestive tract cancer. The median WC (97.0 cm) was set as a reference point according to the guidance [35]. We also modelled the underlying relationship between upper digestive and lower digestive using restricted cubic spline, respectively. Additionally, sensitivity analyses were employed to investigate this association in different subgroups, involving cancer subtypes (upper digestive and lower digestive), BMI (<25, 25-30, 30-35, and ≥35), WC (> median and ≤ median), and sex (male and female) categories. For a more in-depth study of the relationship between WC and digestive tract cancer, we further divided WC into <80 or ≥80 and divided WC according to the presence or absence of abdominal obesity (male: ≥102 and female: ≥88).

Furthermore, we adopted multivariate Cox regression analysis to assess the relationship of WC and BMI with all-cause mortality, and the associations were shown by hazard ratio (HR) with 95% CI. Apart from old age, gender, smoking, drinking, and HbA1c, WC and BMI were also mutually adjusted for. The impact of WC (≤ median and > median) on overall survival for those with digestive tract cancer was illustrated by a Kaplan-Meier curve. We also conducted the same curve by dividing WC into abdominal obesity or not. Statistical significance was defined as a value of *P* < 0.05. R software performed all statistical analyses (version 4.1.2; binary for macOS 11, Big Sur).

### 2.6. Prediction Model

Moreover, on the basis of the analysis we conducted above, a prediction model was accomplished to evaluate the risk of digestive tract cancer. The predictive factors were identified in the logistic regression analysis or in line with references, including waist circumference, body mass index, age, race, gender, education, PIR levels (poverty-income ratio), FPG, smoking, drinking, diabetes, and HbA1c. After eliminating missing values for each variable, 476 samples were eligible. 70% of the samples were used in constructing the model, and the other 30% group served as a validation set. The area under the receiver operating characteristic curve (AUC-ROC) was used to assess the discrimination ability of the predictive model.

## 3. Results

### 3.1. Characteristics of Baseline Data

**Figure 1:**
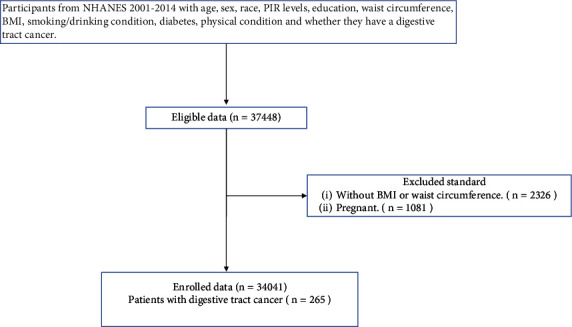
Flow chart of selection of eligible participants. NHANES: National Health and Nutrition Examination Survey.

Of the 37448 individuals aged between ≥18 in the NHANES population, 2326 were excluded due to the lack of a valid BMI or WC and 1081 due to pregnancy. This study eventually involved 34,041 participants, and 265 (0.7%) individuals were diagnosed with digestive tract cancer (Figure 1). Among participants, 50% were female, the median age at inclusion was 46 years (interquartile range, IQR 33–63), and the median WC was 97 cm (IQR 86.8–108). Moreover, the number of participants with abdominal obesity based on WC (male: ≥102 and female: ≥88) was 18359 (53.9%), with 164 of which had digestive tract cancer; while 15658 participants did not have abdominal obesity, the cancer case in which was only 77, showing that abdominal obesity was associated with digestive tract cancer risk (chi-square test, *P* < 0.01). After a median follow-up of 67 months, 97 cases of all-cause death were observed in those with digestive tract cancer, including 31 who died because of cancer-related diseases.

Table 1 summarizes the baseline characteristics of all participants by WC qualities. Compared with the low WC group, the high WC group was older, more male, and less educated. Digestive tract cancer was more observed in the participators with a high WC. Participants with a high WC also had a more possibility to have elevated HbA1c, high FPG, high eGFR, more self-reported diabetes, and more self-reported diseases in the cardiovascular system. Besides, more current smokers and alcoholics were in the low WC and normal WC category than those in the high WC category.

### 3.2. Association of WC with Digestive Tract Cancer

**Figure 2:**
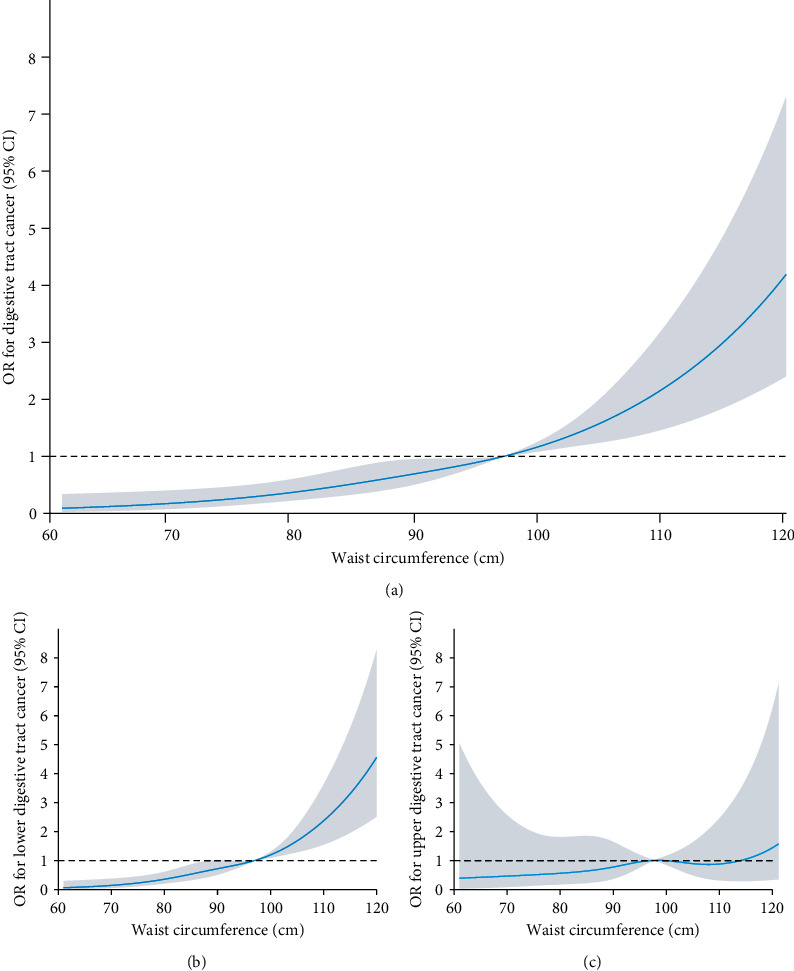
(a) Restricted cubic spline plots of the association between waist circumference and digestive tract cancer. The association was adjusted for age (<80 and ≥80), gender, smoking, drinking, HbA1c, and BMI. The median of the waist circumference was set as the reference for this figure. (b) Restricted cubic spline plots of the association between waist circumference and lower digestive tract cancer. (c) Restricted cubic spline plots of the association between waist circumference and upper digestive tract cancer. BMI: body mass index; CI: Confidence interval.

WC was significantly correlated with the risk of digestive tract cancer (Table 2). In the non-adjusted, minimally adjusted, or fully adjusted models, the ORs with 95% CI were 1.15 (1.07-1.23), 1.13 (1.04-1.22), and 1.72 (1.41-2.10), respectively. After adjusting fully for old age, gender, smoking, drinking, HbA1c, and BMI, individuals with high WC (Q4) had a 2.71-fold increased risk of digestive tract cancer compared to the lowest quartile (Q1). In Figure 2(a), the restricted cubic spline shows a consistent and significant positive association between WC and digestive tract cancer morbidity. However, no significant association was observed between WC and upper digestive cancer (Figure 2(b)). In contrast, lower digestive cancer remained significantly associated with WC (Figure 2(c)).

### 3.3. Association of BMI with Digestive Tract Cancer

In the non-adjusted and minimally adjusted models, there was no significant association between BMI and digestive tract cancer morbidity (OR: 1.01, 95% CI =0.92-1.10; OR: 1.05; and 95% CI =0.95-1.15). However, after fully adjusting the model, the BMI was found to be a protective factor for digestive tract cancer (OR: 0.57 and 95% CI: 0.44-0.73). Moreover, when calculated as a categorical valuable, the OR for BMI were 0.60 (0.41-0.87), 0.47 (0.28-0.79), 0.26 (0.12-0.55), and 0.17 (0.06-0.47) in the overweight, class I obesity, class II obesity, and class III obesity groups. This association was not found in the patients under normal weight (OR: 1.34 and 95% CI =0.40-3.37) (Table 3).

### 3.4. Sensitivity Analysis

**Figure 3:**
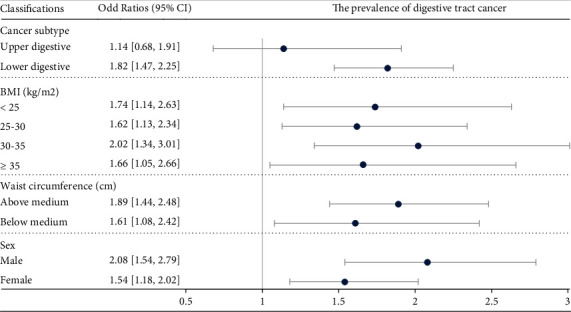
Sensitivity analysis on the association between waist circumference and digestive tract cancer risk based on logistic regression analysis. The association was adjusted for BMI, age, gender, smoking, drinking, and HbA1c. CI: confidence interval; BMI: body mass index.

Figure 3 shows the robust association across cancer subtype (lower digestive), BMI (<25, 25-30, 30-35, and ≥35 kg/m^2^), WC (above median: >97 cm; and below median: ≤97 cm), and sex (male and female). Importantly, when the cut-off WC was set smaller relatively, although a consistent trend remained, WC was not associated with digestive tract cancer risk significantly in the subgroup of WC <80 cm (OR: 1.14 and 95% CI: 0.68-1.91) and participants without abdominal obesity (men: <102 and women: <88) (OR: 1.22 and 95% CI: 0.83-1.80) (Figure S1).

### 3.5. Survival Analysis

**Figure 4:**
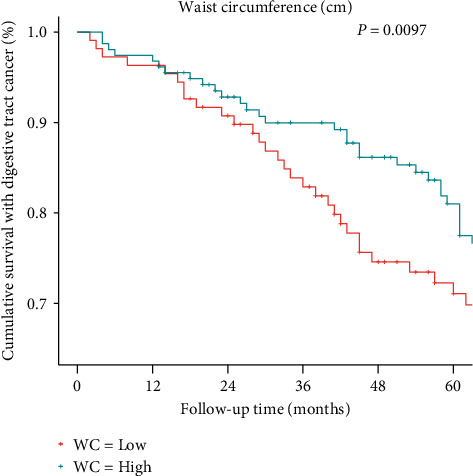
A Kaplan-Meier curve of the association between waist circumference and all-cause mortality of digestive tract cancer in the following 60 months (5 years). The waist circumference was divided by median into two groups (≤97 cm and >97 cm), and the survival comparison among groups was adjusted by the Bonferroni-Holm method. WC: waist circumference.

After adjusting the BMI, WC was associated with a decrement in all-cause mortality (HR: 0.50 and 95% CI: 0.28-0.92). On the contrary, BMI did not show a statistical association once adjusting for the WC (HR: 0.45 and 95% CI: 0.13-1.58) (Table 4). The Kaplan-Meier curve revealed that the survival time was longer among higher WC categories (*P* = 0.0097). Patients whose WC was below or equal to the median (≤97 cm) showed statistically poorer 5-year survival compared with those with a WC above the median (>97 cm) (Figure 4). More importantly, the same conclusion is still met when dividing the WC value according to whether it is consistent with abdominal obesity (abdominal obesity, male: ≥102, and female: ≥88) (Figure S2).

### 3.6. Prediction Model

**Figure 5:**
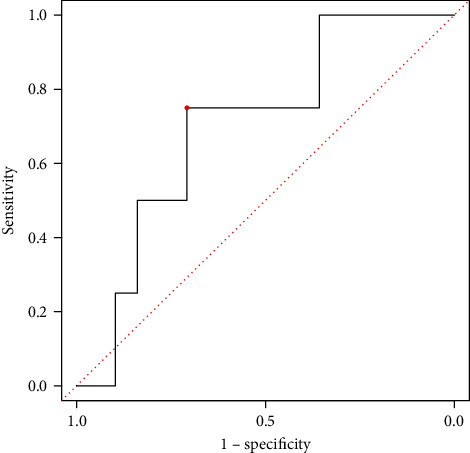
The receiver operating characteristic curve of the identification ability of the proposed nomogram. The nomogram showed relatively strong identification ability with an area under the curve of 0.71 (95% confidence interval, 0.46-0.94), specificity of 0.708, sensitivity of 0.750, and accuracy of 0.972.

As to the model we build, the AUC-ROC of the multivariate logistic regression model to predict the probability of digestive tract cancer was 0.71 (95% CI and 0.46-0.94). Moreover, the specificity of the proposed nomogram was 0.708, the sensitivity was 0.750, and the accuracy was 0.972 (Figure 5).

## 4. Discussion

Accumulating evidence indicates that fat distribution is a primary cause of obesity heterogeneity [36, 37], and abdominal obesity has been recognized as a more serious health problem worldwide, surpassing even obesity defined by BMI [38, 39]. Studies showed that the mean WC of China increased to a greater extent among men and women separately after an adjustment of BMI over 1993-2011 [40]. A similar trend was also observed in the US, England, Mexico, and Canada [40, 41]. Currently, WC is adopted more frequently than BMI or waist-to-hip ratio as the preferred body metric for assessing abdominal fat accumulation, suggesting a more robust association with absolute visceral fat mass [10, 42]. Visceral fat is the underlying culprit for health problems but can only be measured by using expensive instruments directly [43]. In summary, abdominal obesity due to WC significantly increased the adverse health risk, regardless of BMI adjustment.

However, the association between WC and digestive tract cancer now is still vague, and only a few researchers focus on the relationship between WC and digestive tract cancer risk. A recent cross-sectional study reported high WC associated with increased colorectal cancer incidents based on 63057 South Korean population with normal weight [14]. The sample size and selection bias (normal-weight individuals from the health checkup program) made it difficult to generalizable to populations in different settings. In another meta-analysis of prospective studies, Du et al. analyzed the association of WC with total gastroesophageal cancer. It was reported that WC was associated with gastric cancer and esophageal cancer (for gastric cancer, relative risk, *RR* = 1.48 and 95%*CI* = 1.20 − 1.83; for esophageal cancer, *RR* = 2.13 and 95%*CI* = 1.07 − 4.22), while waist-to-hip ratio associated with gastric cancer only (for gastric cancer, *RR* = 1.40 and 95%*CI* = 1.08 − 1.82; for esophageal cancer, *RR* = 2.30 and 95%*CI* = 0.86 − 6.17) [24]. Similarly, Dong et al. [23] performed a meta-analysis on 134,356,0 participants to clarify the association between abdominal obesity and the incidence of colorectal cancer; the relative risk for total colorectal cancer was of more significance in those with greater WC, compared to the low category of WC (*RR* = 1.42 and 95%*CI* = 1.30 − 1.55) [23]. However, few of the included studies in these two meta-analyses studies conducted further adjustments between WC and BMI to clarify their independent role. A consensus statement clearly stated that the robustness of WC and all-cause morbidity can be fully recognized only after adjustment for BMI [10]. Besides, these two meta-analyses did not provide a thorough review of digestive tract cancer.

Consistent with previous studies, we revealed that WC was positively correlated with digestive tract cancer. More importantly, our result ulteriorly showed that the adverse effects of WC persisted even after the adjustment of BMI. Therefore, WC is a strong anthropometric cancer biomarker under any total weight. Those with similar BMI but higher WC represent a phenotype with increased deposition of visceral adipose tissue, rather than the subcutaneous adipose tissue beneath the skin [44]. Visceral adipose tissue is now considered metabolic tissue, playing an important role in immunological, metabolic, and endocrine functions. The pro-inflammatory cytokines (such as tumor necrosis factor-*α*, interleukin-6, and interleukin-1*β*) from visceral adipose tissue contribute to a chronic inflammatory state in the whole body, thus creating a general environment better suitable for tumor growth [45]. As a measurement, WC can be an alternative to visceral adipose tissue [46]. A recent study compared five indicators for measuring visceral fat tissue, demonstrating that WC was plausible to reflect visceral fat accumulation. Unlike WC, BMI is only a weak support measurement of visceral adipose tissue [47]. A preceding study was convinced that abdominal obesity could predict advanced cancer better than BMI [48]. Besides, the WC threshold is currently designed to replace BMI as a proxy for the anthropometric index of obesity without considering its unique advantages in estimating cancer risk. Our findings offered the possibility of establishing a new WC grading scale consistent with BMI, from the perspective of cancer development. Further studies are expected to make this vision a reality.

Also, subgroup analysis in our study showed that WC and the upper digestive tract cancer risk association were not statistically significant. Refined restricted cubic splines of our study showed the same result. The upper digestive tract difference with WC has been reported in previous studies [24, 49, 50]. However, a recent study based on the UK Biobank database conducted a more detailed survey and came to a different result. It reported that WC associated with morbidity of esophageal adenocarcinoma (highest vs lowest category: *HR* = 2.30 and 95%*CI* = 1.47 − 3.57) and with gastric cardia cancer only in men (*HR* = 2.21 and 95%*CI* = 1.27 − 3.84). Besides, it reported that there was no statistically significant association of WC with other tissue types of upper digestive tract cancer, which is in line with the result our article observed [51], reminding us that the histological type of cancer may have an impact on causality.

Moreover, we found that when WC was set to a lower cut-off value in sensitivity analysis, the association between WC and digestive tract cancer became vague. Although these relationships were still approximately positive, their 95% CI continue to widen with the decreasing of cut-off values (Figure 3, Figure s1). Also, Wei et al. conducted a cohort study on 104,825 males in China to clarify the association between WC and primary liver cancer. After a median of 8.9 years of follow-up, they found a U-shaped association between WC and the prevalence of cancer using a restricted cubic spline model (*P*-non-linear = 0.017) [52]. This article reminds us that when the WC is in a low range, the result comes differently. However, all participants in our study came from an American database. Different from the Asian populations, the overall distribution of WC in our study was mostly concentrated in a large value (Figure S3). It is difficult to analyze these changes at lower WCs due to the limited sample in NHANES.

Interestingly, once digestive tract cancer had developed, WC became a protective factor against all-cause mortality in patients (*HR* = 0.50 and 95%*CI* = 0.28 − 0.92). Several studies had similar findings. For example, Lo et al. [53] found that BMI (OR : 0.95 and 95%*CI* = 0.87 to 1.03) and elevated WC (OR : 0.82 and 95%*CI* = 0.67 to 0.99) had an inverse association with cancer-caused mortality. A Cox regression model on 3976 African-American participants in the Jackson Heart Study (JHS) showed a J-shape relationship between WC and overall mortality, after adjusting age, sex, and smoking [54]. Besides, a study investigating the obesity paradox showed that overweight and class I obese (BMI 25-35 kg/m^2^) patients always have a low risk of all-cause mortality after cancer has been diagnosed [55]. However, a recent meta-analysis study found that in women, obesity was associated with higher all-cause and cancer-related mortality in breast cancer [56]. It is of great difference from the findings summarized above, where a high WC appears to be of survival benefit. In summary, during a subset rather than the general population, WC was associated with the decline of all-cause mortality in patients. This result is partially consistent with our research. As a cross-sectional analysis and limited by the NHANES database, we only accumulate data from participants at a certain point in time, ignoring the reverse causation that might exist between WC and the length of survival. Since individuals with cancer have more possibility of a long-time weight loss and decrement of the WC, this limitation may lead to a difference in findings.

Finally, as an extension of previous work, we build a model to evaluate the risk of digestive tract cancers, showing that machine learning algorithms might be a potential tool to predict the occurrence of digestive tract cancers universally, with the factors like waist circumference.

The advantages of this study should be highlighted. Firstly, the database came from the NHANES, a large, constantly updated database. There had a lot of studies using this database to analyze questions, involving cardiovascular diseases, cancers, and metabolic diseases [57–59]. Secondly, previous articles tended to prefer analyzing single cancer and physical indicators such as BMI or WC [60–62]. This article summarized the relationship between cancers that were prone to occur in the entire digestive tract and WC. Thirdly, the association between WC and digestive tract cancer was investigated after adjusting the BMI. This result suggests that WC, independent of BMI, can be a more relevant anthropometric factor than BMI per se. Last but not the least, we made a model to predict cancer happening, showing that machine learning is a tendency in the future to predict digestive tract cancer. There are still some limitations in this study. First, chronic diseases, such as cancer, can cause weight loss [63]. However, due to insufficient data, this article failed to consider the loss of weight caused by long-term chronic diseases. Second, despite the huge size of the NHANES, the amount of upper digestive tract cancer patients in this study was quite small (esophagus cancer: 17, gastric cancer: 26, and total: 43), which may skew the results of this study. The future direction of research will expand the proportion of cancer cases in the total sample size and investigate the relationship between cancer histological types and high WC. Also, most of the cancer data in this article came from the self-reports of patients. There was a possibility that the patient himself was not informed of the condition by the doctor out of humanitarianism. Thirdly, the cross-sectional study of cancer was difficult to judge the causal link. Cohort studies may have a good display of causality; a population-based cohort study that investigated colorectal cancer strongly demonstrated the causality between WC and colorectal cancer [64]. In the next phase of the task, we suggest conducting a cohort study of WC and cancer with a larger database. Finally, previous studies showed that male and female obesity differed in physiology and indicators [65, 66]. Due to the limitation of sample size, this article had not investigated the difference in cancer risk of gender exhaustively and only accrued a subgroup analysis of gender in the sensitivity analysis. Further research is expected. Last but not least, the role of ageing in the association between WC and digestive tract cancer should be further investigated in the following research.

## 5. Conclusions

Measuring WC provides an additional opportunity to improve the estimation of digestive tract cancer, especially cancer located in the lower digestive tract. However, patients with higher WC showed better survival once digestive tract cancer have developed. Further studies should reveal the association between WC and digestive tract cancer and use machine learning methods to predict cancer happening.

## Figures and Tables

**Table 1:** Baseline characteristics divided by quartile of waist circumference.

	Q1 (55.5, 86.8)	Q2 (86.8, 97)	Q3 (97, 108)	Q4 (108, 178)	*P*
*N*	8520	8591	8443	8487	
Digestive tract cancer (%)	45 (0.5%)	64 (0.7%)	70 (0.8%)	86 (1.0%)	0.004
Age (years)	37.0 (25.0, 53.0)	47.0 (34.0, 62.0)	53.0 (39.0, 65.0)	53.0 (39.0, 65.0)	<0.001
Gender (female, %)	5312 (62.3%)	4263 (49.6%)	3690 (43.7%)	3787 (44.6%)	<0.001
Race (%)					<0.001
Non-Hispanic White	3813 (52.1%)	3735 (47.7%)	3988 (49.8%)	4306 (52.4%)	
Non-Hispanic Black	1758 (24.0%)	1661 (21.2%)	1669 (20.8%)	2073 (25.2%)	
Mexican American	1116 (15.2%)	1672 (21.3%)	1685 (21.0%)	1294 (15.8%)	
Other Hispanic	632 (8.6%)	770 (9.8%)	664 (8.3%)	542 (6.6%)	
PIR level (%)					<0.001
<1.33	2550 (29.9%)	2433 (28.3%)	2401 (28.4%)	2627 (31.0%)	
1.33-3.50	2751 (32.3%)	2938 (34.2%)	2895 (34.3%)	2942 (34.7%)	
≥3.50	3219 (37.8%)	3220 (37.5%)	3147 (37.3%)	2918 (34.4%)	
Education (%)					<0.001
Below high school	1906 (22.4%)	2435 (28.4%)	2451 (29.0%)	2349 (27.7%)	
High school	1841 (21.6%)	1914 (22.3%)	2044 (24.2%)	2140 (25.2%)	
Above high school	4760 (56.0%)	4233 (49.3%)	3943 (46.7%)	3995 (47.1%)	
Waist circumference (cm)	80.1 (75.6, 83.6)	92.2 (89.6, 94.6)	102.0 (99.4, 104.8)	116.3 (111.4, 124.3)	<0.001
BMI (kg/m^2^)	22.2 (20.4, 23.9)	26.1 (24.4, 27.8)	29.3 (27.5, 31.4)	35.4 (32.3, 39.7)	<0.001
Esophagus cancer	4	4	6	3	0.765
Stomach cancer	9	7	6	4	0.579
Colon cancer	29	53	57	78	<0.001
Rectal cancer	4	1	3	4	0.566
HbA1c (mmol/L)	5.3 (5.1, 5.5)	5.4 (5.2, 5.7)	5.5 (5.3, 5.9)	5.7 (5.4, 6.2)	<0.001
FPG (mmol/L)	87.0 (81.0, 94.0)	91.0 (85.0, 100.0)	94.0 (87.0, 105.0)	98.0 (89.0, 115.0)	<0.001
Smoking (yes, %)	3508 (41.2%)	3807 (44.3%)	4059 (48.1%)	4352 (51.3%)	<0.001
Drinking (yes, %)	1087 (12.8%)	1099 (12.8%)	1086 (12.9%)	1301 (15.3%)	<0.001
Diabetes (yes, %)	413 (4.8%)	985 (11.5%)	1561 (18.5%)	2574 (30.3%)	<0.001
eGFR (ml/min/1.73m^2^)	95.7 [76.2, 114.3]	102.8 [78.1, 126.0]	108.4 [83.4, 137.1]	133.7 [100.8, 172.5]	<0.001

BMI: body mass index; HbA1c: hemoglobin A1c; FPG: fasting plasma glucose; eGFR: estimated glomerular filtration rate.

**Table 2:** Association of waist circumference with digestive cancer using logistic regression models.

Items	Non-adjusted model		Minimally adjusted model		Fully adjusted model	
	Odds ratio	*P*	Odds ratio	*P*	Odds ratio	*P*
Waist circumference (per 10 cm)	1.15 (1.07-1.23)	<0.001	1.13 (1.04-1.22)	0.002	1.72 (1.41-2.10)	<0.001
Categories						
Q1 (55.5,86.8)	Reference		Reference		Reference	
Q2 (86.8,97)	1.41 (0.97-2.08)	0.076	1.26 (0.86-1.87)	0.239	1.47 (0.98-2.25)	0.082
Q3 (97,108)	1.57 (1.09-2.31)	0.018	1.30 (0.89-1.92)	0.182	1.72 (1.08-2.77)	0.025
Q4 (108,178)	1.93 (1.35-2.79)	<0.001	1.65 (1.14-2.41)	0.009	2.71 (1.48-5.00)	0.001

Minimally adjust model: adjusted for age (<80 and ≥80), gender, smoking, drinking, and HbA1c. Fully adjust model: adjusted for age (<80 and ≥80), gender, smoking, drinking, HbA1c, and BMI.

**Table 3:** Association of BMI with digestive cancer using logistic regression.

Items	Non-adjusted model		Minimally adjusted model		Fully adjusted model	
	Odds ratio	*P*	Odds ratio	*P*	Odds ratio	*P*
BMI (per 5 kg/m^2^)	1.01 (0.92-1.10)	0.835	1.05 (0.95-1.15)	0.359	0.57 (0.44-0.73)	<0.001
BMI categories						
Normal weight	Reference		Reference		Reference	
Underweight	0.87 (0.26-2.10)	0.785	0.79 (0.24-1.92)	0.646	1.34 (0.40-3.37)	0.581
Overweight	1.01 (0.74-1.37)	0.963	0.98 (0.72-1.34)	0.892	0.60 (0.41-0.87)	0.007
Class I obesity	1.14 (0.81-1.60)	0.438	1.17 (0.83-1.66)	0.362	0.47 (0.28-0.79)	0.004
Class II obesity	0.89 (0.53-1.43)	0.642	0.99 (0.58-1.60)	0.961	0.26 (0.12-0.55)	<0.001
Class III obesity	1.03 (0.58-1.71)	0.926	1.22 (0.68-2.08)	0.476	0.17 (0.06-0.47)	<0.001

Minimally adjust model: adjusted for age (<80 and ≥80), gender, smoking, drinking, and HbA1c. Fully adjust model: adjusted for age (<80 and ≥80), gender, smoking, drinking, HbA1c, and waist circumference.

**Table 4:** Association of waist circumference with all-cause mortality of digestive cancer using cox regression model.

Items	Hazard ratio (95% CI)	*P* value
Waist circumference (per 10 cm)	0.50 (0.28-0.92)	0.025
Body mass index (kg/m^2^)	0.45 (0.13-1.58)	0.211

Cox regression model adjusted for age (<80 and ≥80), sex, smoking condition, drinking condition, HbA1c, and interaction between waist circumference and BMI.

## Data Availability

The data used to support the findings of this study comes from National Health and Nutrition Examination Survey (NHANES), a publicly available database in the U.S (https://www.cdc.gov/nchs/nhanes/irba98.htm).

## Supplementary Materials

Supplementary 1 Figure S1. A supplemental sensitivity analysis. The WC was subgrouped into <80 or ≥80 and was subgrouped according to the presence or absence of abdominal obesity (male: ≥102, female: ≥88).

Supplementary 2 Figure S2. A supplemental Kaplan-Meier curve of the association between waist circumference and all-cause mortality of digestive tract cancer in the following 60 months (5 years). The waist circumference was divided by having abdominal obesity or not (male <102 cm, female <88 cm and male ≥102 cm, female ≥88 cm).

Supplementary 3 Figure S3. The overall distribution of waist circumference.
