# Supplementary figures and images for: The Association of Waist Circumference with the Prevalence and Survival of Digestive Tract Cancer in US Adults: A Population Study Based on Machine Learning Methods
Source: Comput Math Methods Med. 2022 Oct 6;2022:2492488. doi: 10.1155/2022/2492488 (PMC9562134; doi:10.1155/2022/2492488)

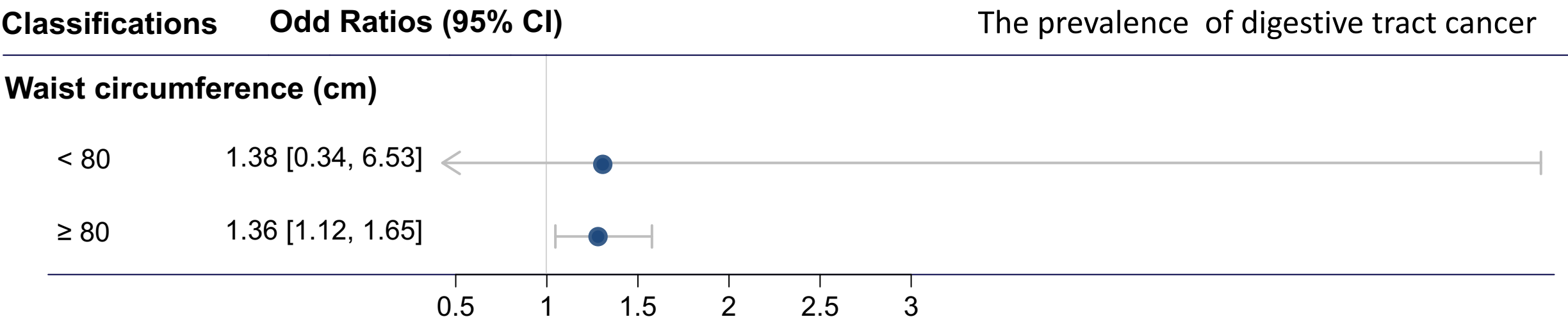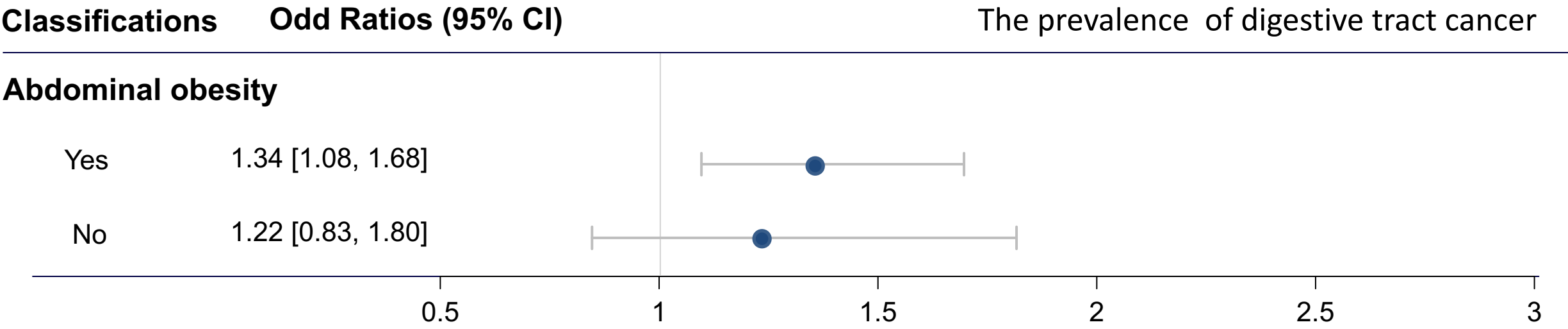

Supplement: Supplementary 1 — Figure S1. A supplemental sensitivity analysis. The WC was subgrouped into <80 or ≥80 and was subgrouped according to the presence or absence of abdominal obesity (male: ≥102, female: ≥88). [file 2492488.f1.pdf]

Waist circumference (cm)

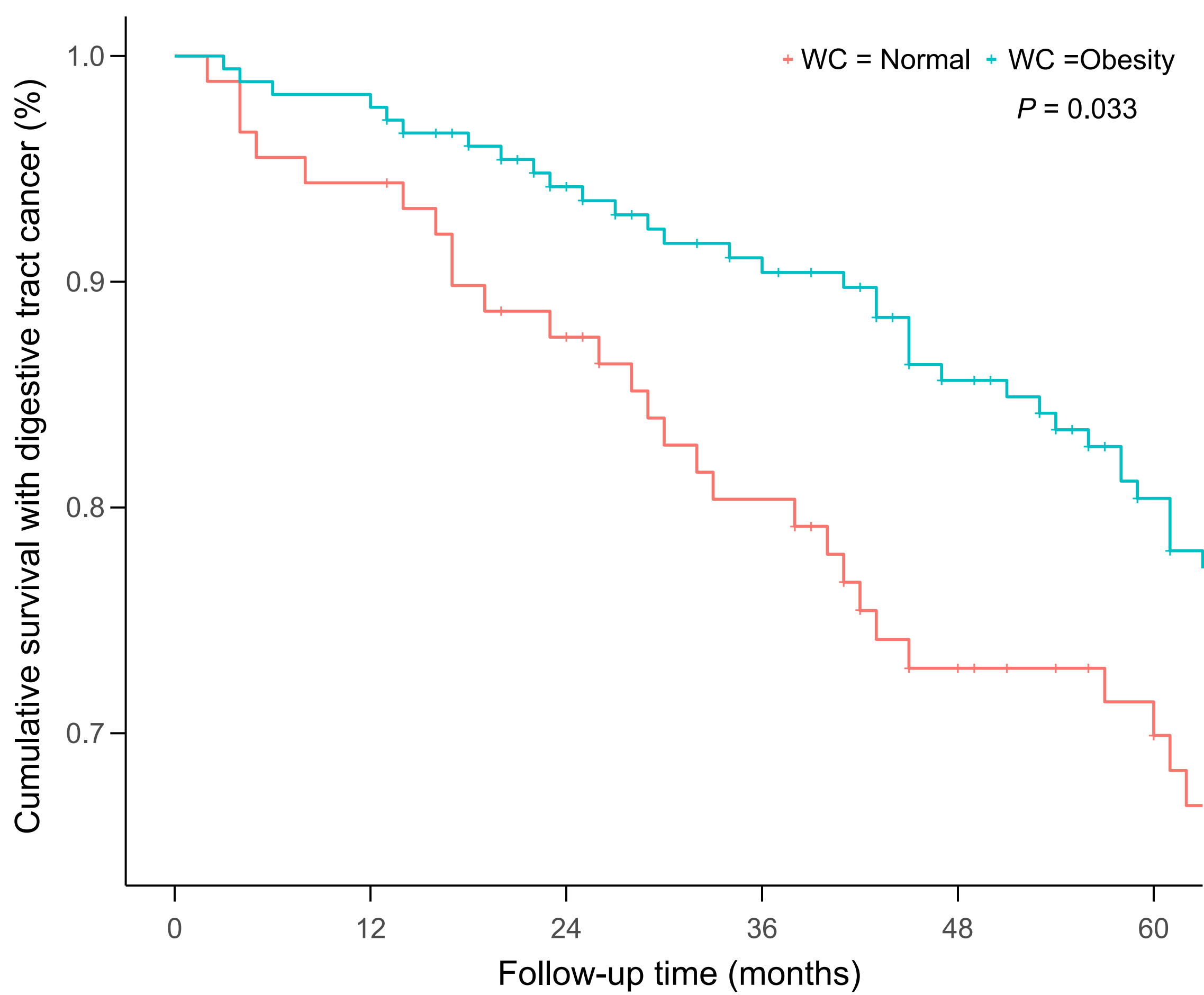

Supplement: Supplementary 2 — Figure S2. A supplemental Kaplan-Meier curve of the association between waist circumference and all-cause mortality of digestive tract cancer in the following 60 months (5 years). The waist circumference was divided by having abdominal obesity or not (male <102 cm, female <88 cm and male ≥102 cm, female ≥88 cm). [file 2492488.f2.pdf]

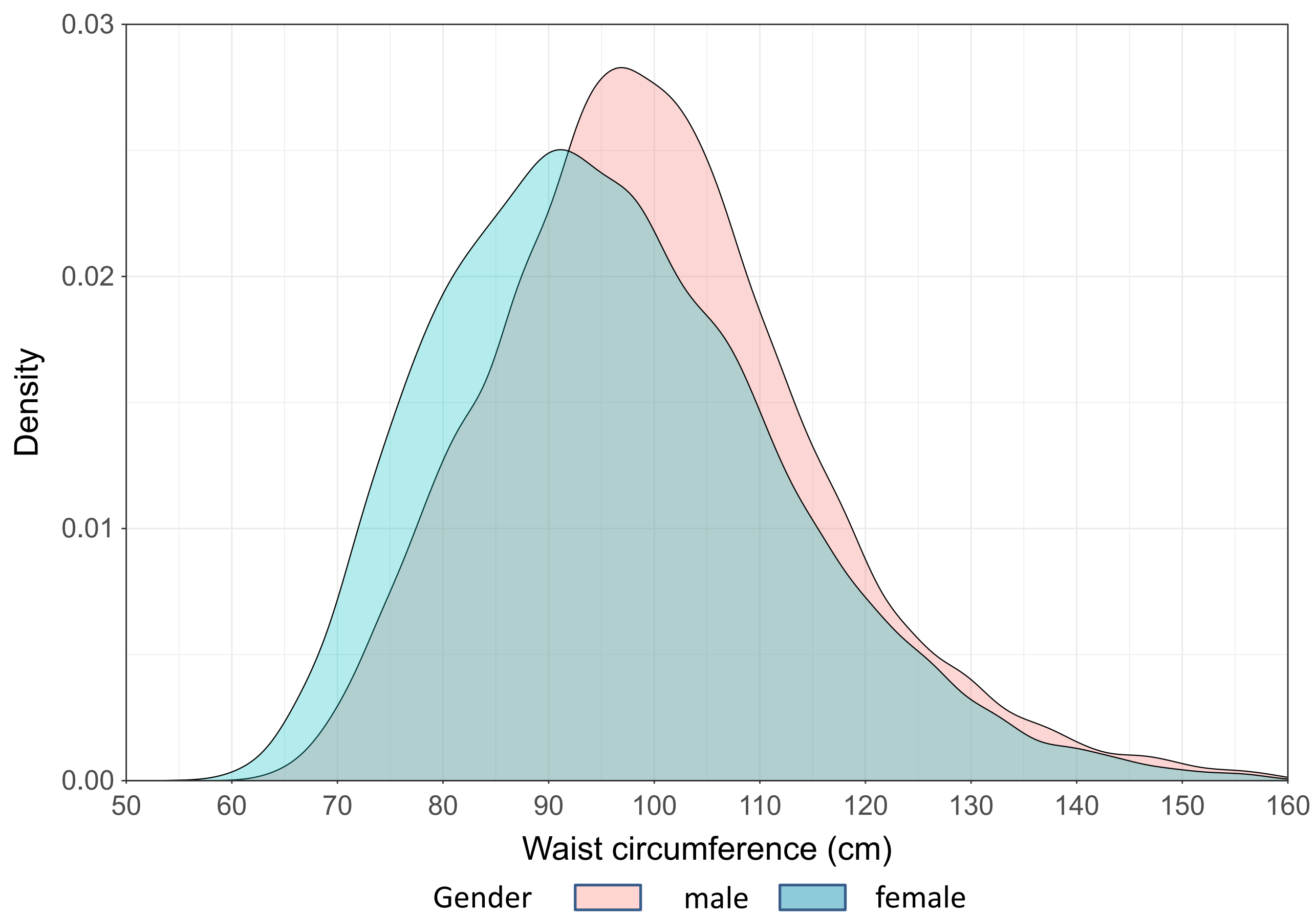

Supplement: Supplementary 3 — Figure S3. The overall distribution of waist circumference. [file 2492488.f3.pdf]
